# A nomogram prediction model for lymph node metastasis risk after neoadjuvant chemoradiotherapy in rectal cancer patients based on SEER database

**DOI:** 10.3389/fonc.2023.1098087

**Published:** 2023-02-27

**Authors:** Xiaoshuang Liu, Li Sha, Cheng Huang, Xiancheng Kong, Feihu Yan, Xiaohui Shi, Xuefeng Tang

**Affiliations:** ^1^ Department of General Surgery, Shuguang Hospital, Shanghai University of traditional Chinese Medicine, Shanghai, China; ^2^ Department of Colorectal Surgery, Shanghai Changhai Hospital, Shanghai, China

**Keywords:** rectal cancer (RC), lymph node metastasis, neoadjuvant chemoradiotherapy (NACRT), nomogram, prediction model, SEER

## Abstract

**Background:**

Rectal cancer patients who received neoadjuvant chemoradiotherapy (CRT) may have a lower cancer stage and a better prognosis. Some patients may be able to avoid invasive surgery. It is critical to accurately assess lymph node metastases (LNM) after neoadjuvant chemoradiotherapy. The goal of this study is to identify clinical variables associated with LNM and to develop a nomogram for LNM prediction in rectal cancer patients following nCRT.

**Methods:**

From 2010 to 2015, patients were drawn from the Surveillance, Epidemiology, and End Results (SEER) database. To identify clinical factors associated with LNM, the least absolute shrinkage and selection operator (LASSO) aggression and multivariate logistic regression analyses were used. To predict the likelihood of LNM, a nomogram based on multivariate logistic regression was created using decision curve analyses.

**Reslut:**

The total number of patients included in this study was 6,388. The proportion of patients with pCR was 17.50% (n=1118), and the proportion of patients with primary tumor pCR was 20.84% (n = 1,331). The primary tumor was pCR in 16.00% (n=213) of the patients. Age, clinical T stage, clinical N stage, and histology were found to be significant independent clinical predictors of LNM using LASSO and multivariate logistic regression analysis. The nomogram was developed based on four clinical factors. The 5-year overall survival rate was 78.9 percent for those with ypN- and 66.3 percent for those with ypN+, respectively (P<0.001).

**Conclusion:**

Patients over 60 years old, with clinical T1-2, clinical N0, and adenocarcinoma may be more likely to achieve ypN0. The watch-and-wait (WW) strategy may be considered. Patients who had ypN0 or pCR had a better prognosis.

## Introduction

In many nations, rectal cancer incidence rates are rising. It is difficult to treat early due to the lack of early clinical symptoms. Many rectal cancer patients are diagnosed at an advanced stage, with local or distant metastases. NCCN (1) and ESMO (2) recommend neoadjuvant chemoradiation (CRT) followed by total mesorectal excision (TME) surgery for locally advanced (T3/4 N0/+M0) rectal cancer. Currently, the lymph node status is crucial for staging, treatment, and prognosis, but the tumor regression grade (TRG) is also a useful marker to evaluate the response to neoadjuvant CRT (3). Many researchers have proposed using local excision or “watch and wait” treatment for rectal cancer patients who have clinically complete response after neoadjuvant CRT (4–6).

Clinical complete response (cCR) indicates that no rectal tumor was discovered during digital examination (DRE), endoscopy, or magnetic resonance imaging (MRI) (7). However, a cCR differs from no pathologic evidence of tumor (pathologic complete response [pCR]), and a pathological assessment can only be performed after TME (8). As a result, accurate assessment of lymph node status prior to surgery is critical for treatment design in rectal cancer patients receiving neoadjuvant CRT. The aim of the study is to investigate the risk factors for lymph node metastasis after neoadjuvant radiotherapy and chemotherapy for rectal cancer, and to develop a predictive model to serve as a guide for patients’ treatment options.

## Patients and methods

### Patient selection

**Figure 1:**
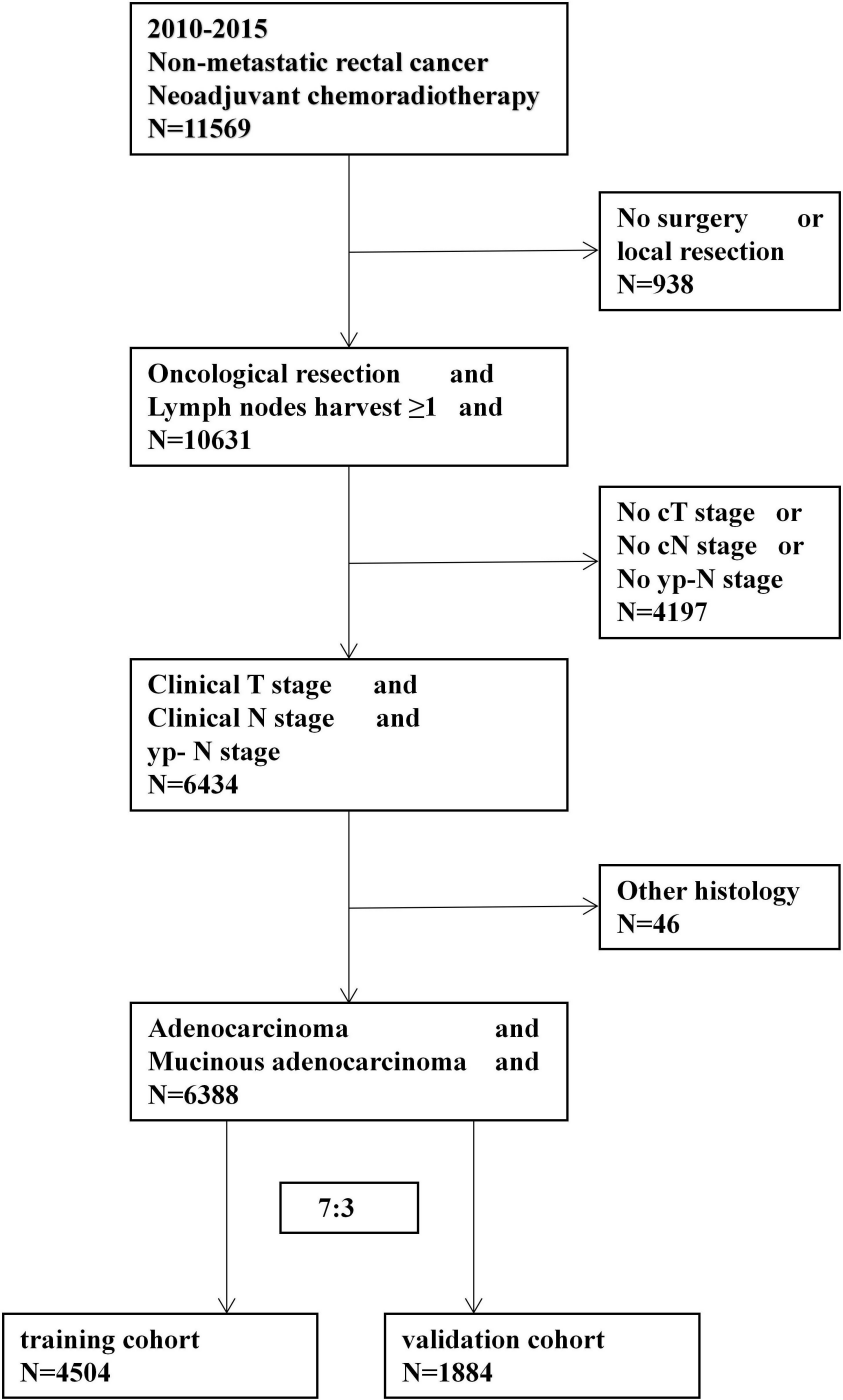
Diagram of patient selection.

SEER*Stat(version 8.3.5) software was used to retrieve information on rectal cancer cases from the surveillance, epidemiology, and end results (SEER) public access database between 2010 and 2015. SEER*Stat is a SEER-provided online program for obtaining patient information. SEER is a representative sample of the US population, with patient-level data collected from 18 geographically diverse populations representing rural, urban, and regional populations (9). The 8th edition of the TNM classification was used to review and stage patients.The selection process is shown in **Figure 1**.

Patients who had received Neoadjuvant CRT after a radical resection were eligible. Patients with no clinical T, clinical N, or ypN stage information and no lymph node harvest were excluded.

### Clinicopathological data

The patient demographics (age, sex, race, year of diagnosis), tumor characteristics (differentiation, clinical T and N stage, ypN stage, tumor histology, tumor size, and pre-treatment CEA level) and survival data were obtained from the SEER database.

### Statistical analyses

The R software (Version 3.6.3; https://www.R-project.org) was used for statistical analysis. Graphpad Prism (Version 6.0) was used to create forest maps and survival graphs.

First, we divided the data at random into training and validation sets in a 7:3 ratio. The least absolute shrinkage and selection operator (LASSO) (10, 11) method, was used to select the potential predictive features. Selected risk factors for lymph node metastasis (LNM) were subjected to multivariate logistic regression analysis (12). The variables with the P ≤ 0.05 were included in the model, whereas tumor size and race were excluded. A nomogram predictive model for LNM following neoadjuvant CRT was created using four variables. Plotting of calibration curves served to evaluate the nomogram’s calibration. The performance of the nomogram’s discrimination was measured using the ROC curve. By measuring the net benefits at various threshold probabilities, decision curve analysis (DCA) was used to assess the nomogram’s clinical applicability. The 5-year overall survival(OS) was estimated by Kaplan-Meier survival curves. Log-rank tests were conducted to assess statistical significance.

## Results

### Patient characteristics

This study enrolled a total of 6388 rectal cancer patients. The proportion of patients with pCR was 17.50% (n=1118), with primary tumor pCR being 20.84% (n = 1,331). While the primary tumor was pCR, 16.00% (n=213) of the patients were N+. The training cohort consisted of 4504 patients, while the validation cohort consisted of 1884 patients. LNM was found in 27.4% (1232/4504) of the training cohort and 27.2% (513/1884) of the validation cohort. Preoperative clinical factors including age, race, sex, CEA, tumor size, clinical T stage, clinical N stage, Year of diagnosis, grade and histology were shown in **Table 1**. Six potential predictors were chosen from the training cohort’s ten clinicopathological factors.

**Table 1:** Patient characteristics.

Characteristics	The training cohort	The validation cohort
**Number of patients**	4504	1884
Age (years)		
<40	224	44
40-60	2059	219
≥60	2221	220
Clinical T stage		
cT_1/2_	579	250
cT_3/4_	3925	1634
Clinical N stage		
cN_0_	2166	891
cN_+_	2338	993
Race		
White	3731	414
Black	356	44
Other	408	54
Unknown	9	1
Sex		
Male	2769	318
Female	1735	195
Year of diagnosis		
2010	163	77
2011	184	68
2012	191	74
2013	209	104
2014	248	87
2015	237	103
Tumor size		
<3cm	136	55
3-5cm	351	147
≥5cm	568	234
Unknown	177	77
CEA (ng/ml)		
Negative	496	185
Positive	409	183
Unknown	327	145
Histology		
Adenocarcinoma	1154	460
Mucinous adenocarcinoma	78	53
Grade		
Well differentiation	70	30
Moderate differentiation	847	341
Poor differentiation	142	68
Undifferentiated	26	10
Unknown differentiation	147	64

### Risk factors for LNM

**Figure 2:**
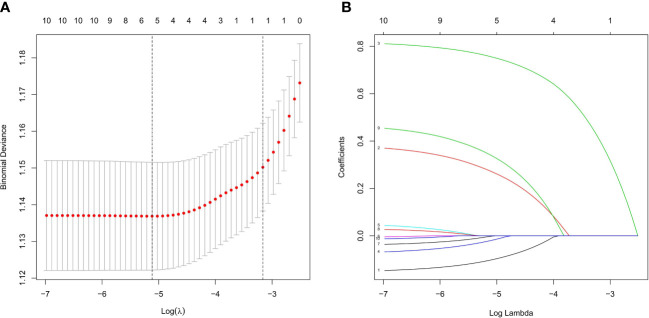
Feature selection using LASSO logistic regression. **(A)** Tuning parameter (l) selection in the LASSO logistic regression used 10-fold cross-validation *via* minimum criteria. The binomial deviance was plotted versus log (λ). The black vertical lines were plotted at the optimal λ based on the minimum criteria and λ standard error of the minimum criteria. **(B)** LASSO coefficient profiles of the 10 clinical factors. A coefficient profile plot was produced versus the log (λ).

**Figure 3:**
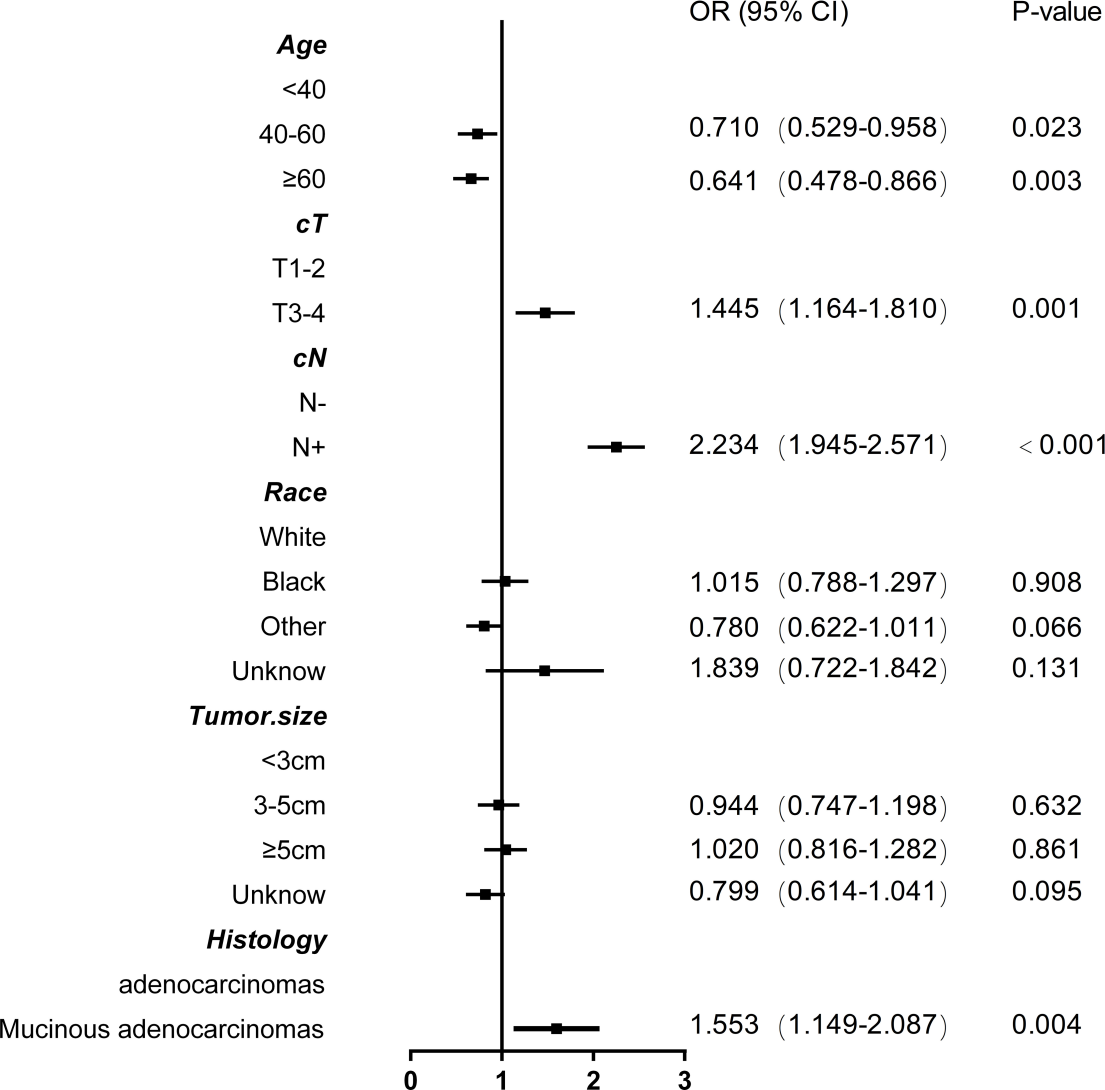
Forest plot with odds ratios base on multivariable logistic model associations with lymph node metastasis in rectal cancer patients after neoadjuvant chemoradiotherapy.

Based on nonzero coefficients in the LASSO logistic regression model, risk factors for LNM in rectal cancer patients receiving neoadjuvant CRT were identified using the LASSO method and multivariate logistic regression models (**Figure 2**). The six potential predictors were further evaluated using a multivariate logistic regression model to optimize the predictive model. Age (P<0.05), clinical T stage (P<0.001), clinical N stage (P<0.001), and histology (P=0.004) were found to be independent LNM risk factors for rectal cancer patients receiving neoadjuvant CRT by the multivariate logistic regression analysis (**Figure 3**).

### Development and validation the prediction model

**Figure 4:**
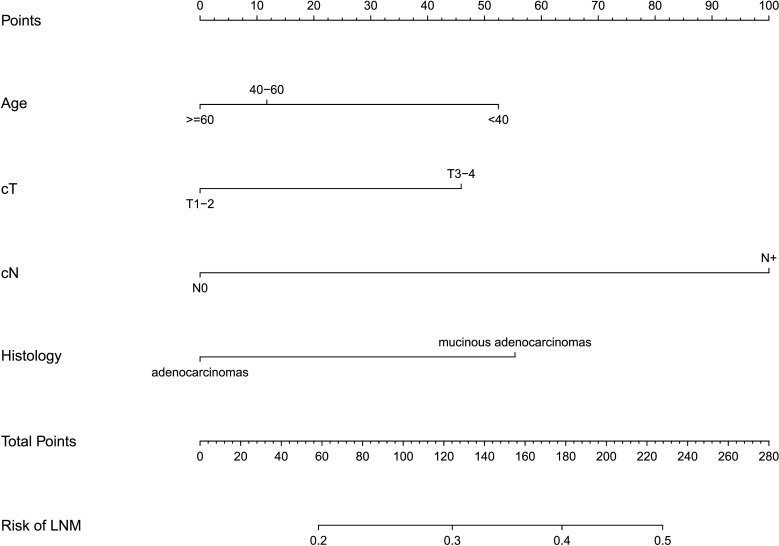
The nomogram for predicting lymph node metastasis in rectal cancer patients after neoadjuvant chemoradiotherapy. The nomogram was established in the training cohort using multivariable regression, consisting of age, cT(clinical T stage), cN(clinical T stage)and histology.

Based on the four independent risk factors (age, clinical T stage, clinical N stage, and histology), a nomogram was developed (**Figure 4**). A vertical line is drawn for each variable to see their respective score when using. The total score, which determines the risk probability of LNM, was calculated by adding each score together. For example, a 50- year-old patient (points= 12) with T3/T4 classification (points=46), with mucinous adenocarcinomas (points=55), with clinical N stage positive (points=100)would have a total score of 213, and a predicted LNM risk of 45%.

**Figure 5:**
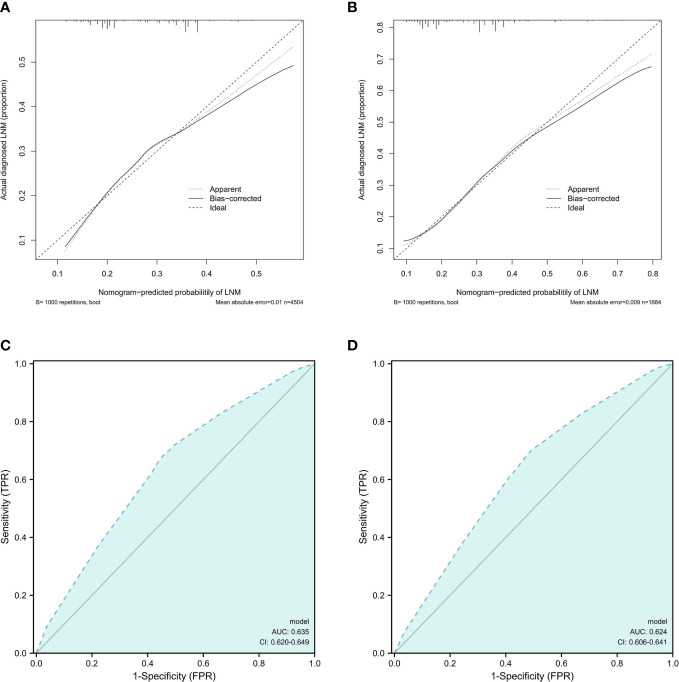
Calibration curves and ROC curves analysis. **(A, B)**.Calibration curves for LNM rates predicted by nomogram for training and validation cohorts **(C, D)**. Receiver operating characteristic (ROC) curves of the nomogram of LNM; The AUC values of the ROC curve predicted LNM rates for the nomogram in the training cohort, and validation cohort.

As shown in **Figure 5**, the calibration curve showed that the training and validation cohort curves were both close to the 45-degree line, indicating that the model can perfectly predict real events. The prediction model’s area under the ROC curve (AUC) for the training cohort was 0.635 (95% CI: 0.620-0.649).We used internal validation to test and verify the nomogram. The validation cohort’s calibration curve and ROC curve produced similar results to the training cohort. The area under the ROC curve (AUC) for the prediction model’s validation cohort was 0.624 (95% CI: 0.606-0.641).

### Clinical use and prognosis

**Figure 6:**
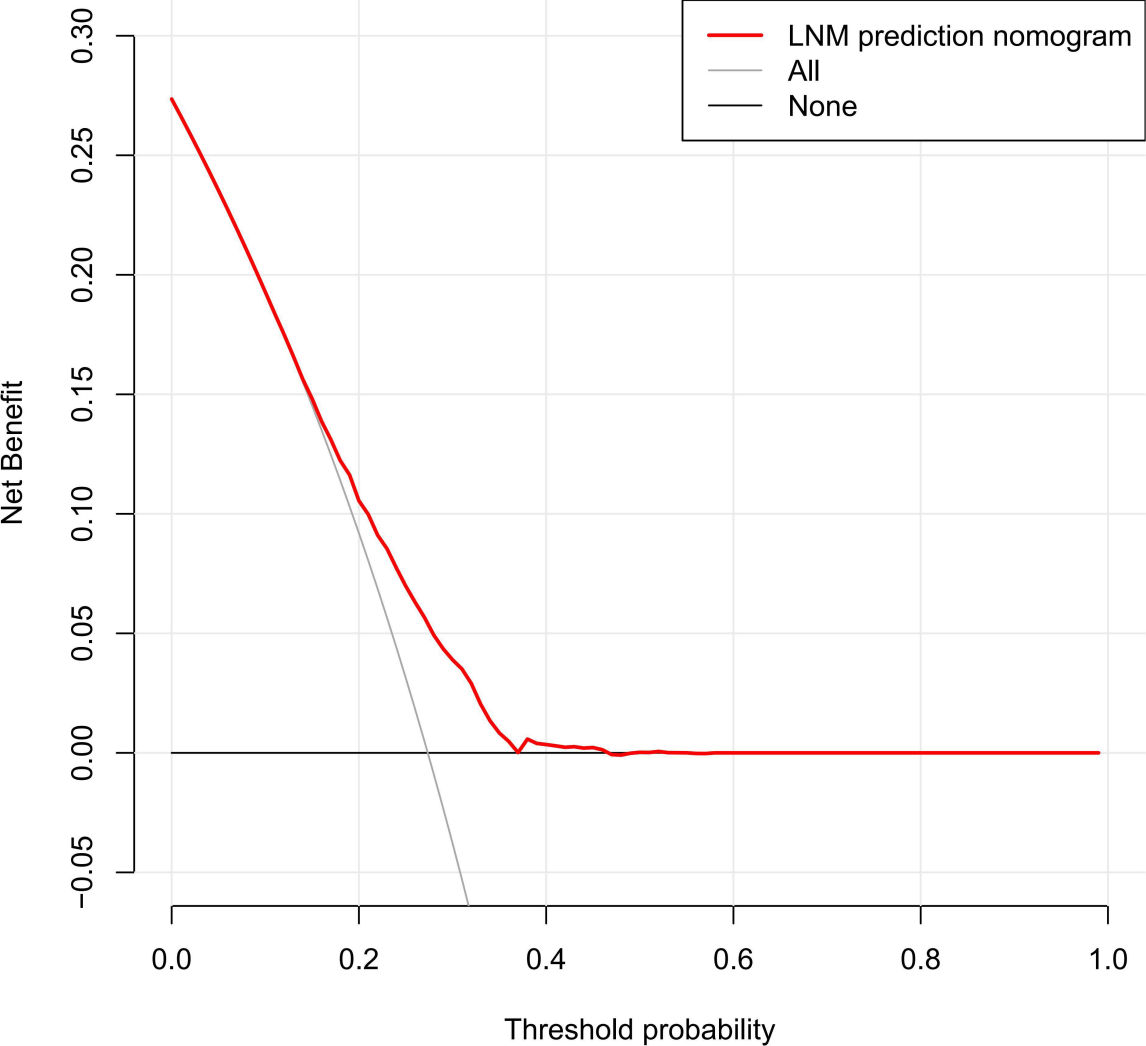
Decision curve analysis.

**Figure 6** depicts the decision curve analysis for the nomogram. The decision curve analysis revealed that clinical decisions were superior to a scenario in which all patients or none were treated across a wide range of thresholds ranging from 0.10 to 0.46.

**Figure 7:**
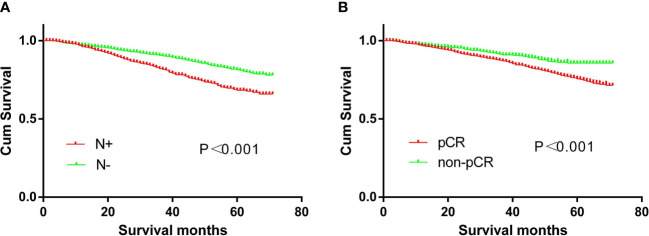
Survival curves. **(A)** The prognosis of CRC patients with or without N+ (p<0.01). **(B)** The prognosis of CRC patients with or without pCR (p<0.01).

The 5-year overall survival of the N+ and N- groups is 66.3% and 78.9%, respectively (p<0.001), while the non-PCR and PCR groups are 75.2% and 85.7%, respectively (p<0.001) (**Figure 7**).

## Discussion

Currently, neoadjuvant chemoradiotherapy is the pre-operative standard of care the for locally advanced rectal cancer. A promising non-operative management, the “watch and wait” approach, was first proposed by Dr. Angelita Habr-Gama for patients achieving clinical complete response following chemoradiotherapy (4). This approach avoids major surgical trauma, preserves anal function, and significantly improves the quality of life. Thus, the research interest has rapidly grown (8, 13, 14) and several clinical trials are ongoing (NCT03561142, NCT04246684). Julio Garcia-Aguilar and colleagues recently found that total neoadjuvant therapy (employing intensive schedules) may help half of the patients achieve organ preservation (14). Noteworthy, novel systemic agents are paving the way to futuristic non-operative management, also avoiding standard neoadjuvant treatments in selected clusters of patients (15). At present, there is currently no method for accurately predicting lymph node metastases after radiotherapy and chemotherapy. Residual metastatic lymph nodes may be the main reason for treatment failure in some patients. According to some studies, cCR does not equal pCR (16–18), and pCR of primary tumor does not equal complete lymph node remission. Patients with primary tumor pCR still have a 12.6-17.4% lymph node metastasis rate (17, 19).

Nomograms have been widely used to visualize colorectal cancer risk factors and prognosis (20–22). The nomogram is a graphical computational scale, and the minimum absolute shrinkage and selection operator (LASSO) is a regression analysis method. The combination of these two approaches aids in the quantification of individual risks for specific outcomes in various cancers (23). Few studies have used nomogram to predict lymph node metastasis following neoadjuvant chemoradiotherapy.

Our study evaluated the risk factors for lymph node metastasis after neoadjuvant chemoradiotherapy for rectal cancer, and a nomogram was used to visualize which patients were more likely to develop lymph node metastasis. Ten risk factor candidates were chosen in our study to form a nomogram by narrowing down the regression coefficients using the lasso method, which has been recommended for variable selection (20, 24). The best predictors were then identified using a multivariate logistic regression model (25, 26). Finally, four potential predictors nomograms were established.

According to the findings, younger patients, particularly those under the age of 40, have a higher proportion of lymph node metastases after neoadjuvant chemoradiotherapy. According to the findings of a few non-neoadjuvant chemoradiotherapy studies, the risk factors for lymph node metastasis in colorectal cancer patients, the younger patients had a higher risk of lymph node metastasis (27–29). Other research has found that the number of lymph nodes detected in colon cancer is related to age, with younger patients having a higher number of lymph nodes harvested (30, 31). A Chinese study also found that the risk of lymph node metastasis increased with age (32). However, a Germany study found that age was not related to lymph node metastasis following rectal cancer radiotherapy and chemotherapy (33).

The study founded that the higher the T stage after neoadjuvant chemoradiotherapy for rectal cancer, the greater the risk of lymph node metastasis. This was consistent with our usual clinical perception. According to a meta-analysis, higher T stage was associated with a higher rate of lymph node metastasis (34). Wang et al. found that the risk of lymph node metastasis following neoadjuvant chemoradiotherapy was related to T stage, with the deeper the invasion, the greater the risk of lymph node metastasis (32).

MRI is the most commonly used imaging tool for assessing lymph node metastasis prior to treatment, and it has a high level of accuracy. It is obvious that clinical N stage is related to postoperative lymph node metastasis. This was consistent with our findings; a multi-center study in the Netherlands found that cN+ was associated with lymph node metastasis following neoadjuvant chemoradiotherapy (19). In the meantime, our study found that patients with rectal signet ring cell carcinoma had a higher risk of lymph node metastasis following neoadjuvant chemoradiotherapy. This was consistent with the findings of several studies. The more poorly differentiated patients in the Netherlands study also had higher lymph node metastasis after neoadjuvant chemoradiotherapy (19). Wang et al. found that the risk of lymph node metastasis after neoadjuvant chemoradiotherapy was related to differentiation (32).

In our study, patients with pCR had a 5-year overall survival rate of 85.7%. The survival was not poor, but the prognosis was poor when compared to other studies, in which the 5-year overall survival of pCR was 93% (35) and 94% (8), respectively. This could be due to the SEER database’s incomplete preoperative chemoradiotherapy. Furthermore, the data included ranged from 2010 to 2015, and chemoradiotherapy was inconsistent during this time period. Furthermore, the median patient age in both studies was less than 60 years, at 57 years (8) and 59 years (35), respectively.

The 5-year overall survival rate of patients with non-pCR in our study was 75.2%, and a study using National Cancer Database (NCDB) data showed a survival rate of 73% (35), which was similar to our findings. Meanwhile, a meta-analysis found that patients with ypN0 and ypN+ disease had 5-year overall survival rates of 83.2% and 63.4%, respectively (34). The rate was also comparable to our study, which was 78.9% and 66.3%, respectively.

However, there were some limitations. Firstly, because the study was conducted retrospectively, the level of evidence was low. Secondly, the study was based on data from the SEER database, and the dose and timing of radiotherapy and chemotherapy were unknown. Thirdly, the SEER database does not specify the examination method used for preoperative staging, which may cause some inaccurate staging. Finally, the study used internal verification rather than external data for verification.

In conclusion, we developed a four-risk nomogram for predicting LNM in CRC patients who received CRT. Lymph nodes are still more likely to be positive after CRT in young, pre-treatment cN+, cT3/4, or mucinous adenocarcinoma patients. Strategies such as radical surgery rather than local resection or “watch-and-wait” may be appropriate.

## Data availability statement

The raw data supporting the conclusions of this article will be made available by the authors, without undue reservation.

## Ethics statement

The studies involving human participants were reviewed and approved by Shuguang Hospital. The patients/participants provided their written informed consent to participate in this study.

## Author contributions

XL, LS, and CH contributed equally to this work. XL, FY, and XS contributed to the study design and literature search. XL, LS, CH, and XK collected and analyzed the data. XL, LS, and CH contributed to the literature search and the writing of the manuscript. FY, XS, and XT contributed to the assessment of literature quality, review and revise of the manuscript. FY, XS, and XT are the correspondent authors. All authors contributed to the article and approved the submitted version.
